# Changing the spin disorder of two-dimensional magnetic Cr_2_TiC_2_T_x_ to long-range order through noble metal adhesion

**DOI:** 10.1016/j.isci.2024.109227

**Published:** 2024-02-16

**Authors:** Jianhui Yang, Fei Shi, Huaiyuan Zhao, Liang Chen

**Affiliations:** 1 Quzhou University, Quzhou 324000, P.R. China; 2 Key Laboratory of Advanced Fuel Cells and Electrolyzers Technology of Zhejiang Province, Ningbo Institute of Materials Technology and Engineering, Chinese Academy of Sciences, Ningbo, Zhejiang 315201, P.R. China

**Keywords:** Natural sciences, Physics, Condensed matter physics, Magnetism

## Abstract

To enhance the use of Cr2TiC2Tx MXene in spin electronics, it is essential to transform its spin-disordered state into a long-range ordered spin state. In this study, first-principles calculations show that Rh layers adhered to the Cr_2_TiC_2_T_x_ surfaces can transform its spin disordered state into a long-range spin order by donating electrons to the O terminations, resulting in Cr_2_TiC_2_T_x_ becoming a single-layer A-type antiferromagnet. As the proportion of F termination increases from 0 to 100%, the exchange coupling constant J_1_ of the compound escalates from 0.5 to 15.9 meV. Concurrently, the Néel temperature experiences a significant rise from 8 K to 110 K. The analysis of the density of states reveals that the obtained Cr_2_TiC_2_T_x_ exhibits excellent conductivity with O termination and semiconductor characteristics with F termination. These unique features make Cr_2_TiC_2_T_x_ a promising magnetic material for application in spin electronics.

## Highlights

• Rh layers can transform spin disordered Cr_2_TiC_2_T_x_ into A-type antiferromagnet
• Proportion of F termination increases the Néel temperature of Cr_2_TiC_2_T_x_ to 110 K
• O-terminated Cr_2_TiC_2_T_x_ is conductive while F-terminated is semiconductor

## Graphical abstract

**Figure:**
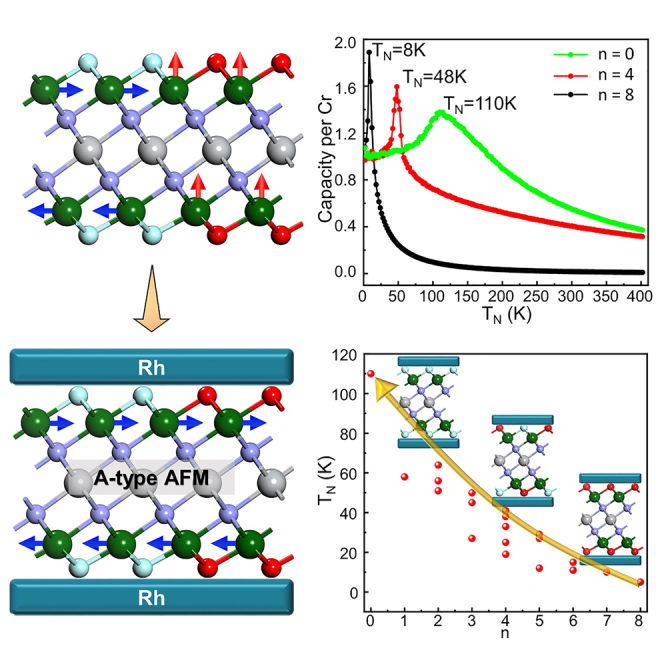

## Introduction

Antiferromagnetic (AFM) materials do not exhibit macroscopic magnetization as the magnetic moments of adjacent magnetic atoms have opposite directions; they can thus withstand magnetic-field interference to a larger extent than ferromagnetic (FM) materials.^1^^,^^2^^,^^3^^,^^4^^,^^5^ Besides, the strong AFM coupling between magnetic moments in AFM materials can result in a resonance frequency in the terahertz (THz) range.^6^^,^^7^ Their high-speed intrinsic spin-wave modes at THz frequencies endow them with the ability to carry information with low loss and high-speed propagation. Thus, AFM materials have unique advantages for application to spin electronic devices, magnetic storage, etc., which can promote the development of 6G communication.^8^^,^^9^^,^^10^^,^^11^ Investigating the magnetic properties of AFM materials and their application to spin electronic devices has attracted increasing attention.^1^^,^^12^^,^^13^ In recent years, the technology for manipulating magnetic states of AFM has been improved thanks to the efforts of researchers around the world. For example, it has been found that the magnetic order can be tuned through external factors, such as electricity, light.^4^^,^^14^ The spin direction of AFM materials can be manipulated by heating, applying an external magnetic field, or applying an electric current or an electric field to the system. It can also be tuned through the electrode change induced by the piezoelectric effect.^15^

Many studies have indicated that two-dimensional (2D) magnetic materials can be used to prepare ultrathin spin-electronic devices and realize ultra-compact spintronics.^16^^,^^17^ In contrast to traditional bulk materials, 2D materials are mechanically flexible, and their magnetic properties can be easily tuned through surface engineering. Many different 2D AFM materials have been synthetized, which can be broadly classified into the three following categories: zigzag, Néel, and A-type AFM materials.^16^ Most 2D A-type AFM materials investigated so far, such as CrI_3_ and VSe_2_, exhibit intralayer ferromagnetic (FM) and interlayer AFM couplings in their bilayer form.^18^^,^^19^ Through density functional theory calculations, Cr_2_TiC_2_F_2_, which is a 2D Cr-based MXene, was predicted to be a single-layer A-type AFM material with superior thermoelectric performance and high magnetoresistance.^20^^,^^21^^,^^22^ This single-layer A-type AFM material can have high spin-wave frequencies due to its strong exchange coupling between two magnetic layers.^7^^,^^23^ Recently, stringent computational studies have confirmed that bare Ti_2_C and Cr_2_CCl_2_ MXene also exhibits characteristics of an A-type AFM material.^24^^,^^25^^,^^26^

Recently, Gogotsi et al. synthesized the magnetic Cr-based MXene (Cr_2_TiC_2_T_x_), in which the terminations (T_x_) are a mixture of F and O in varying ratios.^27^ However, the results from field-cooled (FC) and zero field-cooled (ZFC) magnetization experiments suggest a spin-glass state with a magnetic phase transition temperature of 30 K. In this spin-glass state, ferromagnetic and AFM domains are randomly distributed, and the directions of the magnetic moments are randomly frozen, exhibiting long-range disorder. The dominant AFM interactions were confirmed by the negative Weiss temperature. Such a spin disorder can be attributed to the nonuniform mixture of the O and F terminations. Cr_2_TiC_2_O_2_ is a ferromagnet, while Cr_2_TiC_2_F_2_ is an antiferromagnet.^28^ The terminations of Cr_2_TiC_2_T_x_ MXene after being etched by hydrofluoric acid are F, OH, and O; they are distributed nonuniformly,^27^^,^^28^^,^^29^ which leads to the spin-glass state.

To promote the application of Cr_2_TiC_2_T_x_ MXene in spintronics, a long-range spin order is required. Hence, it is crucial to be able to change the spin disordered state of single-layer Cr_2_TiC_2_T_x_ MXene to long-range spin order. In this study, 4d noble metal (Rh, Pd, and Ag) layers were deposited on the Cr_2_TiC_2_T_x_ MXene surfaces in order to tune its magnetic properties. First-principles calculation results show that the adhesion of the Rh and Pd layers to the Cr_2_TiC_2_T_x_ surfaces changes the spin disordered state of Cr_2_TiC_2_T_x_ to long-range spin order with single-layer A-type AFM arrangement.

## Calculation models

**Figure 1:**
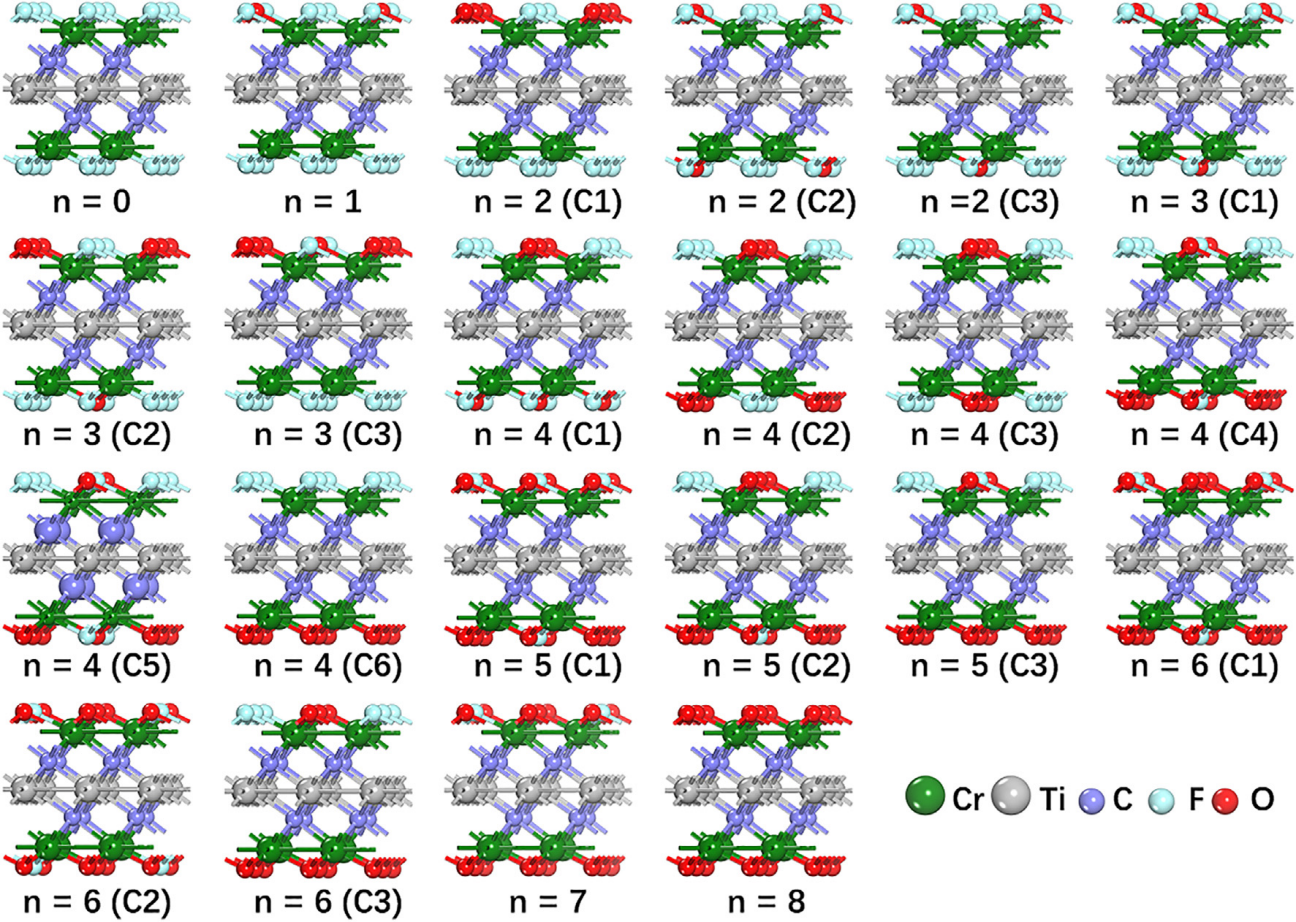
Side views of different F and O adsorption configurations for Cr_2_TiC_2_O_n/4_F_2−n/4_ with n = 0, 1, 2, 3, 4, 5, 6, 7, and 8

Cr_2_TiC_2_T_x_ MXene is mainly terminated by F and O atoms.^29^ Thus, Cr_2_TiC_2_O_n/4_F_2−n/4_ (n = 0, 1, 2, 3, 4, 5, 6, 7, and 8) models similar to previously reported models in the literature were used in the present study,^28^ and the same adsorbing configuration for the F and O terminations was considered as shown in Figure 1.

**Figure 2:**
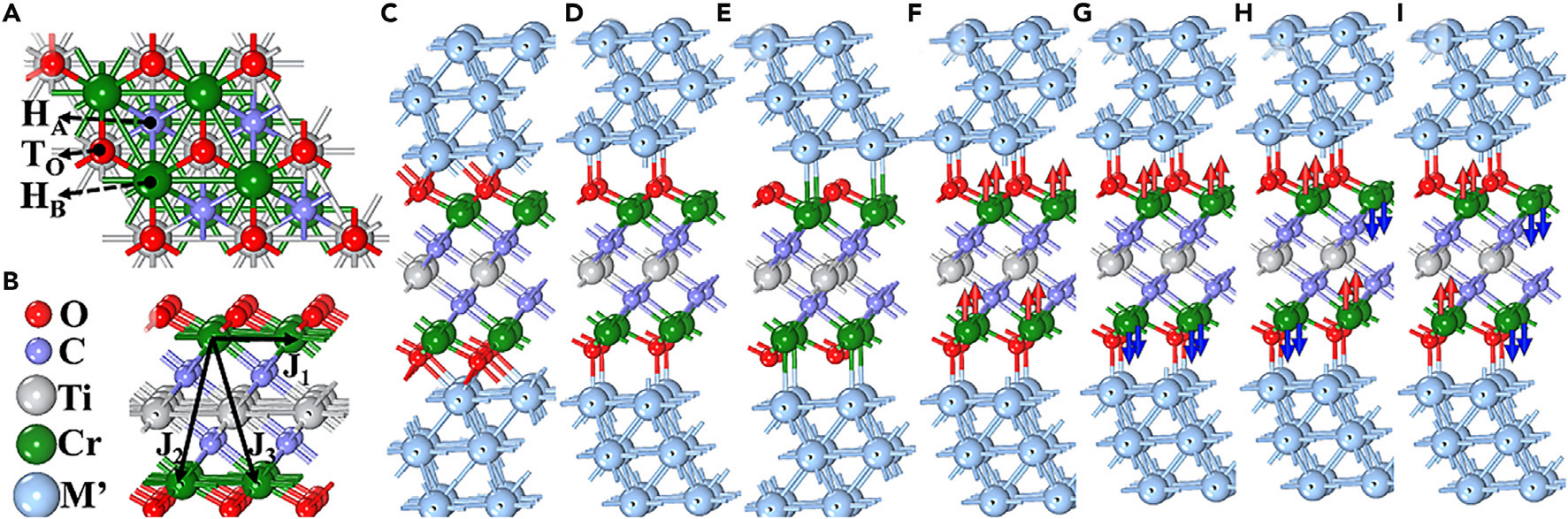
Top and side views for simulated models (A) and (B) Top and side view of the 2 × 2 Cr_2_TiC_2_O_2_ supercell, where the three possible adsorption sites for the noble metals are indicated. (C–E) Side views of the three different M/Cr_2_TiC_2_O_n/4_F_2−n/4_/M heterojunction models investigated. (F–I) Side views of the (F) FM, (G) AFM1, (H) AFM2, and (I) AFM3 arrangements of the M/Cr_2_TiC_2_O_n/4_F_2−n/4_/M heterojunctions.

Cr_2_TiC_2_T_x_ MXene was sandwiched between noble metal layers to form an M/Cr_2_TiC_2_O_n/4_F_2−n/4_/M (M = Ph, Pd, and Ag) heterojunction. The three noble metal layers were made to adhere to both Cr_2_TiC_2_O_n/4_F_2−n/4_ surfaces. Our previous works showed that the strain of MXene may cause an FM–AFM phase transition.^22^^,^^30^ Therefore, to avoid the influence of external strain on the spin arrangement, the optimized lattice of pure Cr_2_TiC_2_O_n/4_F_2−n/4_ was used, and it was fixed in the heterojunctions. For the adsorption of the noble metal layers, three adsorption sites with high symmetry were considered, as shown in Figure 2A, where a 2 × 2 supercell Cr_2_TiC_2_O_2_ is taken as an example. These three sites are the top O atom site (T_O_), hollow A-site (H_A_) with a C atom below it, and hollow B-site (H_B_) with a Cr atom below it. The M/Cr_2_TiC_2_O_n/4_F_2−n/4_/M heterojunctions were modeled with three atomic layers both above and below the H_A_, T_O_, and H_B_ sites, the side views of which are illustrated in Figures 2C–2E. The FM and three AFM arrangements (AFM1, AFM2, and AFM3) shown in Figures 2F–2I were considered.

## Results and discussion

### Magnetic arrangements of the M/Cr_2_TiC_2_O_n/4_F_2−n/4_/M heterojunctions

Firstly, we need to determine the adsorption configurations of the M/Cr_2_TiC_2_O_n/4_F_2−n/4_/M heterojunctions. The calculation results show that the noble metals are preferentially adsorbed on the T_O_ site. For models in which the noble metals are adsorbed on the H_A_ and H_B_ sites, the noble metals are then transferred to the T_O_ site upon structural optimization. This result is similar to our previous finding, according to which Au atomic layers are also preferentially adsorbed on the O top sites of the Cr_2_NO_2_ surface.^17^ Thus, in the following, the model in which the noble metals are adsorbed on the T_O_ site for the M/Cr_2_TiC_2_O_n/4_F_2−n/4_/M heterojunctions is used.

The energies of the FM and three AFM arrangements of M/Cr_2_TiC_2_O_n/4_F_2−n/4_/M were compared to find the ground state. For convenience, the energy differences of the AFM1 (ΔE_AFM1_), AFM2 (ΔE_AFM2_), and AFM3 (ΔE_AFM3_) states with respect to the FM state for the different M/Cr_2_TiC_2_O_n/4_F_2−n/4_/M systems were calculated. If all the ΔE_AFM_ are positive, M/Cr_2_TiC_2_O_n/4_F_2−n/4_/M prefers to be in the FM state. Previous research works have shown that ΔE_AFM1_ increases from −0.18 to 0.29 eV as n increases from 0 to 8 for pure Cr_2_TiC_2_O_n/4_F_2−n/4_,^28^ which indicates that Cr_2_TiC_2_O_n/4_F_2−n/4_ prefers to be in the AFM1 state in the case of a low O coverage, while it prefers to be in the FM state in the case of a high O coverage. Owing to the inhomogeneous O distribution and coverage, Cr_2_TiC_2_T_x_ MXene presents a spin-glass state.

**Figure 3:**
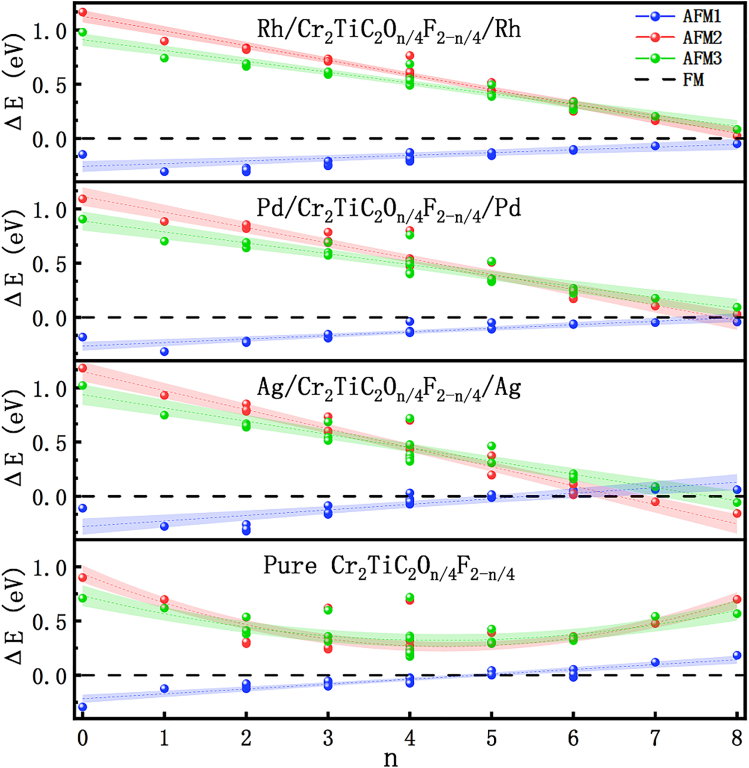
Energy difference of the AFM1 (ΔE_AFM1_), AFM2 (ΔE_AFM2_), and AFM3 (ΔE_AFM3_) states with respect to the FM state for the different M/Cr_2_TiC_2_O_n/4_F_2−n/4_/M heterojunctions

Similar to pure Cr_2_TiC_2_O_n/4_F_2−n/4_, ΔE_AFM1_ increases as n increases for the M/Cr_2_TiC_2_O_n/4_F_2−n/4_/M heterojunctions, as shown in Figure 3. By contrast, ΔE_AFM1_ remains negative for the Rh/Cr_2_TiC_2_O_n/4_F_2−n/4_/Rh and Pd/Cr_2_TiC_2_O_n/4_F_2−n/4_/Pd heterojunctions as n increases from 0 to 8. This indicates that the M/Cr_2_TiC_2_O_n/4_F_2−n/4_/M (M = Pd or Rh) heterojunctions prefer the AFM1 arrangement regardless of the degree of coverage and distribution of the O terminations. This prevents the occurrence of the magnetic phase transition caused by the F and O terminations and can thus lead to a long-range spin order for these two heterojunctions. Specifically, the adhesion of the Rh and Pd layers causes a magnetic phase transition for Cr_2_TiC_2_O_2_ (n = 8) from the FM to the AFM1 state. From the point of view of ΔE_AFM1_, the adhesion of the Rh layers is better than that of the Pd layers, which benefits the formation of a stable, long-range spin order for the AFM1 arrangement. In contrast to the Rh and Pd layers, the adhesion of the Ag layers causes a magnetic phase transition for Cr_2_TiC_2_O_2_ from the FM to the AFM2 state. Thus, Ag/Cr_2_TiC_2_O_n/4_F_2−n/4_/Ag prefers to be in the AFM1 (AFM2) state for a low (high) O coverage. This also results in the Ag/Cr_2_TiC_2_O_n/4_F_2−n/4_/Ag heterojunction exhibiting the spin-glass state.

In order to investigate the spin order of the M/Cr_2_TiC_2_O_n/4_F_2−n/4_/M heterojunctions, the spin direction of the Cr atoms was determined by calculating the magnetic anisotropy energy (MAE) according to the following equation:

$$\text{M}\text{A}\text{E}=\;\left({\text{E}}_{001}\;-{\;\text{E}}_{100}\;\right)/{\text{N}}_{\text{C}\text{r}}\text{,}$$

where E_100_ and E_001_ are the total energies of the M/Cr_2_TiC_2_O_n/4_F_2−n/4_/M heterojunctions with the spin of the Cr atoms along the [001] and [100] directions, respectively. N_Cr_ is the number of Cr atoms. A negative value of the MAE indicates that the magnetic easy axis of the Cr atoms is along the c-axis, while a positive value indicates that the magnetic easy axis is in the xy-plane.

**Figure 4:**
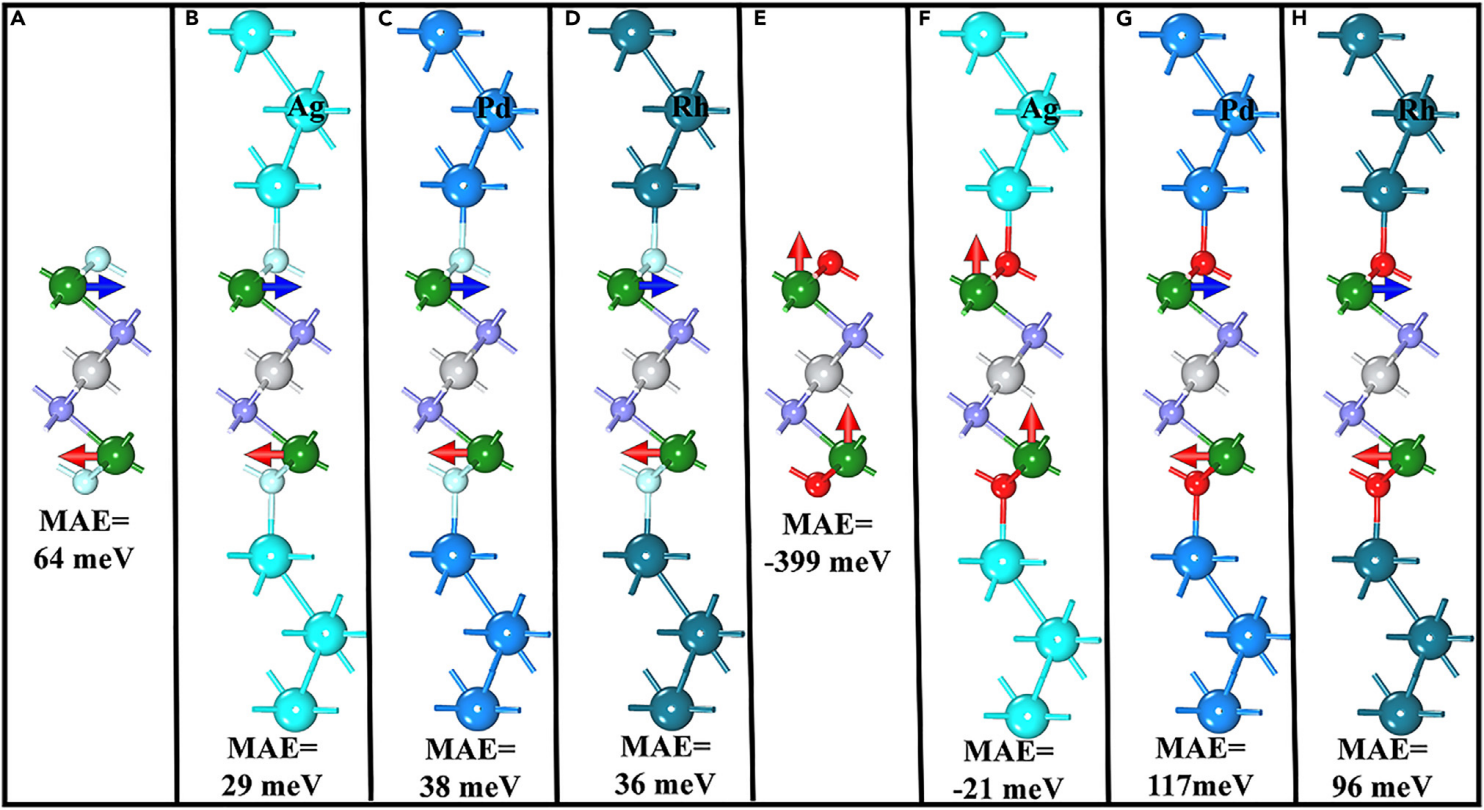
MAE values and spin directions of the different M/Cr_2_TiC_2_F_2_/M and M/Cr_2_TiC_2_O_2_/M heterojunctions (A) and (E) represent for pure Cr_2_TiC_2_F_2_ and Cr_2_TiC_2_O_2_ respectively. (B–D) represent for Ag/Cr_2_TiC_2_F_2_/Ag, Pd/Cr_2_TiC_2_F_2_/Pd, Rh/Cr_2_TiC_2_F_2_/Rh respectively. (F–H) represent for Ag/Cr_2_TiC_2_O_2_/Ag, Pd/Cr_2_TiC_2_O_2_/Pd, Rh/Cr_2_TiC_2_O_2_/Rh respectively.

To understand the effect of the metal layer adhesion on the spin direction of Cr_2_TiC_2_T_x_ MXene, the MAE values of the M/Cr_2_TiC_2_O_2_/M and M/Cr_2_TiC_2_F_2_/M systems were calculated and analyzed. Pure Cr_2_TiC_2_F_2_ favors the AFM1 arrangement with the spins lying in the xy-plane, as shown in Figure 4. Such a spin arrangement remains unchanged even when the noble metal layers adhere to the Cr_2_TiC_2_F_2_ surfaces, while the MAE value decreases slightly from 61 meV (for pure Cr_2_TiC_2_F_2_) to 29 (for M = Ag), 38 (for M = Pd), and 34 meV (for M = Rh). Different from the case of Cr_2_TiC_2_F_2_, the adhesion of noble metal layers causes a marked variation in the spin arrangement of Cr_2_TiC_2_O_2_. The ground state of Cr_2_TiC_2_O_2_ is FM with the magnetic easy axis perpendicular to the noble metal layers. However, the adhesion of the Rh and Pd layers causes the ground state to exhibit the AFM1 arrangement with the magnetic easy axis in the xy-plane. Thus, M/Cr_2_TiC_2_O_2_/M ends up having the same spin arrangement as M/Cr_2_TiC_2_F_2_/M, finally leading to the long-range spin order for Cr_2_TiC_2_T_x_ MXene. The adhesion of the Ag layers does not induce any change in the direction of the magnetic easy axis. However, it makes Cr_2_TiC_2_O_2_ favor the AFM2 state, which is different from the preferred state of Cr_2_TiC_2_F_2_.

### Magnetic properties of the Rh/Cr_2_TiC_2_T_x_/Rh heterojunction

**Figure 5:**
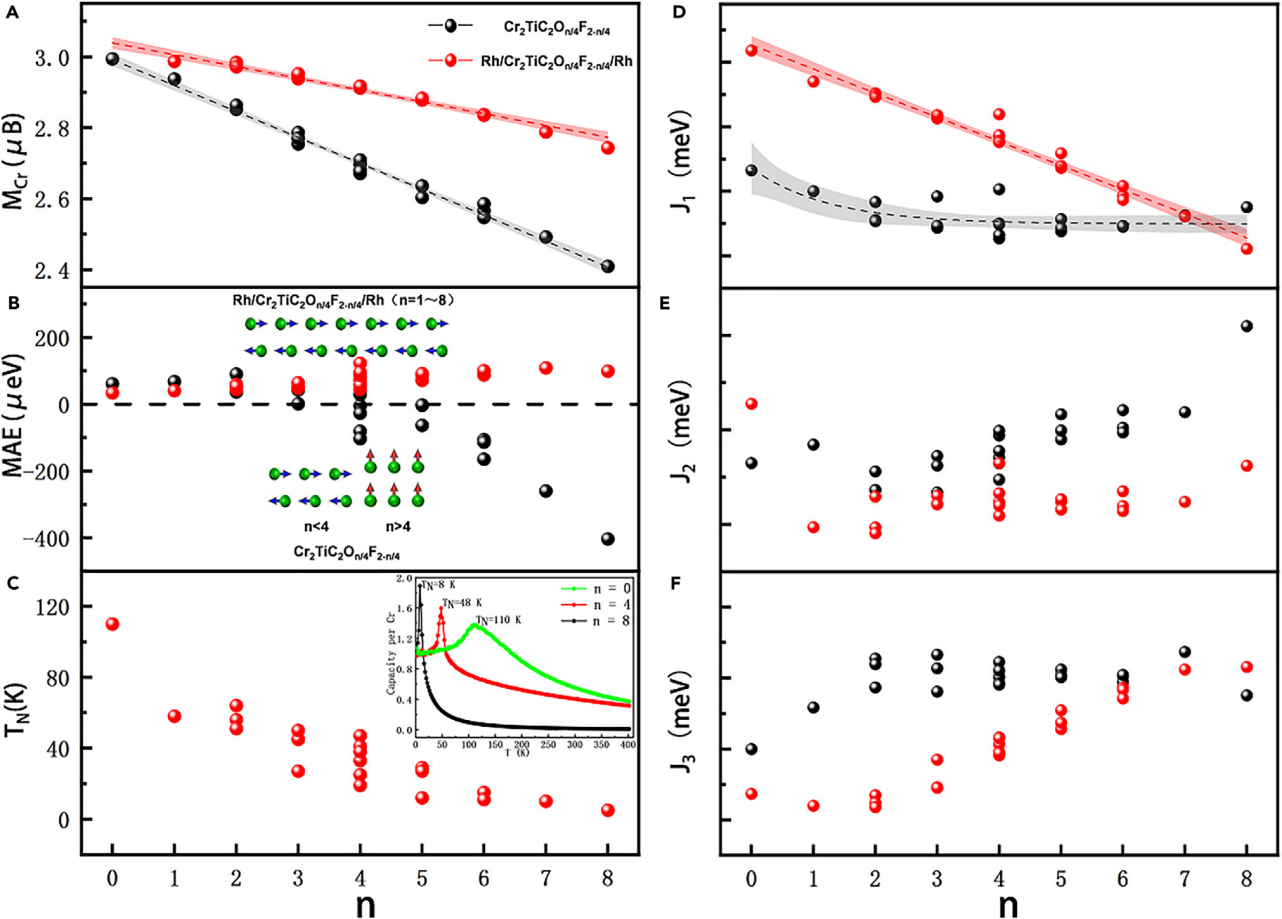
M_Cr_, MAE, T_N_, and J values of Rh/Cr_2_TiC_2_O_n/4_F_2−n/4_/Rh as a function of n (A–C) M_Cr_, MAE and T_N_ for Rh/Cr_2_TiC_2_O_n/4_F_2−n/4_/Rh. (D–F) J_1_, J_2_, and J_3_ for Rh/Cr_2_TiC_2_O_n/4_F_2−n/4_/Rh.

As mentioned before, the Rh layers are the most effective in inducing a long-range spin order for the AFM1 arrangement. The magnetic properties of the Rh/Cr_2_TiC_2_O_n/4_F_2−n/4_/Rh heterojunction were analyzed in detail. The average magnetic moment of the Cr atoms (M_Cr_) decreases as n increases, as shown in Figure 5A. Such a trend is also similar to that of pure Cr_2_TiC_2_O_n/4_F_2−n/4_. On the other hand, the adhesion of the Rh layers leads to an increase in M_Cr_. For the Cr_2_TiC_2_O_1.25_F_0.75_ (n = 5) system, the O coverage is similar to that reported experimentally for the Cr_2_TiC_2_O_1.3_F_0.8_ structure,^27^^,^^29^ and the theoretical value of M_Cr_ is 2.64 μ_B_.^28^ As the Rh layers adhere to its surfaces, the M_Cr_ value becomes 2.88 μ_B_, which corresponds to an increase of about 9.1%.

For pure Cr_2_TiC_2_O_n/4_F_2−n/4_, the MAE value is positive for n < 3, and it becomes negative for n > 5, as shown in Figure 5B. This means that the magnetic easy axis is in the xy-plane for a low O coverage, while it is along the c-axis for a high O coverage. This inhomogeneous O coverage is what causes the spin disorder for Cr_2_TiC_2_O_n/4_F_2−n/4_. However, the MAE value remains positive when the Rh layers adhere to the Cr_2_TiC_2_O_n/4_F_2−n/4_ surfaces regardless of the value of n (between 0 and 8). This indicates that the adhesion of the Rh layers to the Cr_2_TiC_2_O_n/4_F_2−n/4_ surfaces can cause the spins to lie in the xy-plane and form a long-range spin arrangement even in the presence of an inhomogeneous O coverage.

Monte Carlo (MC) simulations were used to estimate the Néel temperature (T_N_) of the Rh/Cr_2_TiC_2_O_n/4_F_2−n/4_/Rh heterojunctions; these simulations were implemented using the MCSOLVER package.^31^ The corresponding exchange coupling parameters (J_1_, J_2_, and J_3_) were calculated through the Heisenberg model. Here, J_1_, J_2_, and J_3_ represent the nearest, next-nearest, and next-next-nearest neighbor exchange coupling parameters, as shown in Figure 2B. The number of Cr ions with the same/opposite spin direction with respect to the nearest, next-nearest, and next-next-nearest neighbor Cr ions do not change in the presence of the Rh layers. Thus, the Heisenberg–Hamiltonian equations for the J_1_, J_2_, and J_3_ parameters of the Rh/Cr_2_TiC_2_O_n/4_F_2−n/4_/Rh heterojunctions are the same as those of the pure Cr_2_TiC_2_O_n/4_F_2−n/4_ systems, as it has been shown in a previous study.^28^

As shown in Figure 5C, the T_N_ of the Rh/Cr_2_TiC_2_F_2_/Rh heterojunction (i.e., for n = 0) is up to 110 K. However, T_N_ decreases as n increases: It is about 20–50 K for n = 4 and decreases to 8 K for n = 0. Nonetheless, recent studies have pointed out that there exist several methods for increasing the T_N_ or Curie temperature (T_C_) of 2D magnetic materials, such as electron doping.^32^ Thus, manipulating the magnetic properties of these single-layer A-type AFM materials may bring novel results and spintronic applications.

Similar to T_N_, the exchange coupling parameter J_1_ also decreases as n increases, as shown in Figure 5D. It reaches the minimum value for the Rh/Cr_2_TiC_2_O_2_/Rh heterojunction with n = 8. Such a decrease of J_1_ is the main factor behind the decrease of T_N_, as J_1_ represents the exchange coupling between each pair of nearest-neighbor Cr ions. However, J_1_ remains positive as n increases from 0 to 8. This leads to each pair of nearest-neighbor Cr ions of the Rh/Cr_2_TiC_2_O_n/4_F_2−n/4_/Rh heterojunctions having the same spin direction, and thus the material is a single-layer A-type antiferromagnet. In contrast to J_1_, the values of J_2_ and J_3_ can be both positive and negative as n increases from 0 to 8, as shown in Figures 5E and 5F.

### Electronic mechanism behind the variation of the magnetic properties

**Figure 6:**
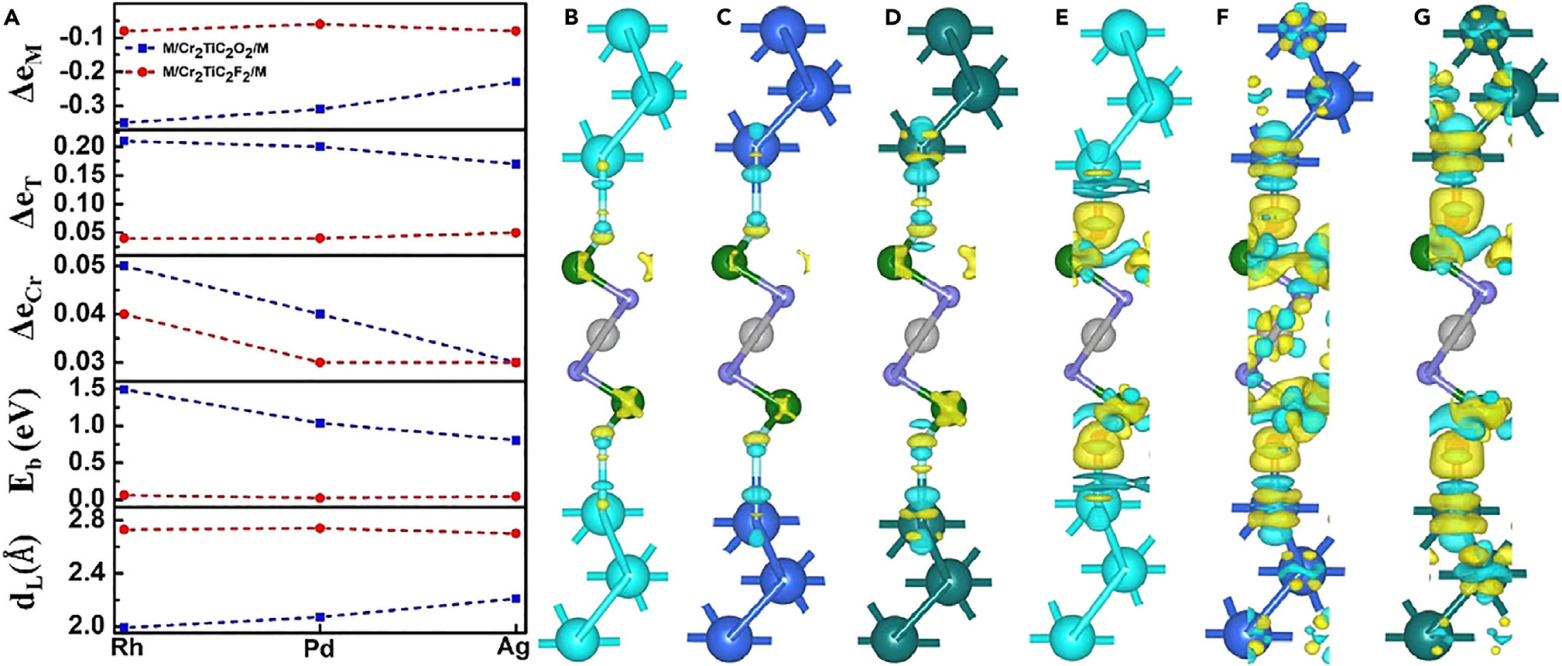
Charge transfer and E_b_, d_L_ in M/Cr2TiC2O2/M and M/Cr2TiC2F2/M heterojunctions (A) Δe_M_, Δe_T_, Δe_Cr_, E_b_, and d_L_ of the M/Cr_2_TiC_2_O_2_/M and M/Cr_2_TiC_2_F_2_/M heterojunctions. Visualization of the charge transfer in the (B) Ag/Cr_2_TiC_2_F_2_/Ag, (C) Pd/Cr_2_TiC_2_F_2_/Pd, (D) Rh/Cr_2_TiC_2_F_2_/Rh, (E) Ag/Cr_2_TiC_2_O_2_/Ag, (F) Pd/Cr_2_TiC_2_O_2_/Pd, and (G) Rh/Cr_2_TiC_2_O_2_/Rh heterojunctions. The yellow and blue isosurfaces correspond to electron accumulation and depletion, respectively. The isosurface levels are set to 0.002 e/Å^3^.

The electron transfer (Δe) between noble metals and Cr_2_TiC_2_T_x_ MXene was analyzed to investigate the interaction in the heterojunctions. Here, we use Cr_2_TiC_2_O_2_ and Cr_2_TiC_2_F_2_ as two typical representatives of Cr_2_TiC_2_T_x_ MXene. The Bader charge analysis was performed to investigate the electron transfer in the heterojunctions. A positive value of Δe represents an electron gain, while a negative value represents an electron loss. Overall, the electrons are transferred from the noble metals to Cr_2_TiC_2_T_x_ MXene as the heterojunctions are formed. For the M/Cr_2_TiC_2_O_2_/M heterojunctions, Δe_M_ ranges between −0.23 and −0.35. It indicates that the electrons move from the noble metal to MXene. The absolute values of Δe_M_ of the M/Cr_2_TiC_2_O_2_/M heterojunctions are obviously higher than those of the M/Cr_2_TiC_2_F_2_/M heterojunctions, as shown in Figure 6A. Such difference in Δe_M_ is mainly caused by the extra unoccupied orbital of the O atoms with respect to the F atoms. The majority of the transferred electrons occupy the orbitals of the O atoms, while the remaining electrons occupy the orbitals of the Cr atoms.

According to Anderson’s theoretical analysis,^33^ when the number of electrons is less than that required to fill the shells to half, the FM arrangement is favored; however, when the number of electrons is higher, the AFM arrangement is preferred. It is these electrons that are transferred from the noble metals to MXene that cause the FM-to-AFM1 phase transition in Cr_2_TiC_2_O_2_ and hence give rise to the long-range spin order of Cr_2_TiC_2_T_x_ when the distribution of the O and F terminations on its surfaces is inhomogeneous. Among the three noble metals, Rh has the greatest ability to donate electrons, as shown in Figures 6B–6G. Hence, the long-range spin order is more easily induced in the Rh/Cr_2_TiC_2_O_n/4_F_2−n/4_/Rh heterojunctions than in the other heterojunctions.

The binding energy (E_b_) and the layer distance (d_L_) between Cr_2_TiC_2_T_x_ and the noble metal layers were also calculated to analyze the interaction between the two material systems. Specifically, d_L_ is the average distance between the F or O terminations and the nearest noble metals along the c-axis. E_b_ was calculated as follows:

$$E_{b}=\left[E\left(M\right)+E\left({Cr}_{2}TiC_{2}O_{n/4}F_{2-n/4}\right)–E\left(Total\right)\right]/\left(2\times N\right)\text{,}$$

where E(Total) is the total energy of the M/Cr_2_TiC_2_O_n/4_F_2−n/4_/M heterojunction, E(M) and E(Cr_2_TiC_2_O_n/4_F_2−n/4_) are the total energies of the individual layers in the same supercell of the corresponding structures, and N is the number of M atoms in one layer. The factor 2 in the denominator stems from the fact that each supercell has two identical interfaces.

The E_b_ of the M/Cr_2_TiC_2_F_2_/M heterojunctions is in the range of 0.02–0.09 eV per M atom. Such a low E_b_ and high d_L_ indicate that the interaction between M and Cr_2_TiC_2_F_2_ is a van der Waals interaction. This can be attributed to the inertness of the F atoms. The E_b_ of the M/Cr_2_TiC_2_O_2_/M heterojunctions is in the range of 0.8–1.5 eV per M atom. This moderate interaction does not destroy the Cr_2_TiC_2_T_x_ structure but has an influence on its magnetic properties.

**Figure 7:**
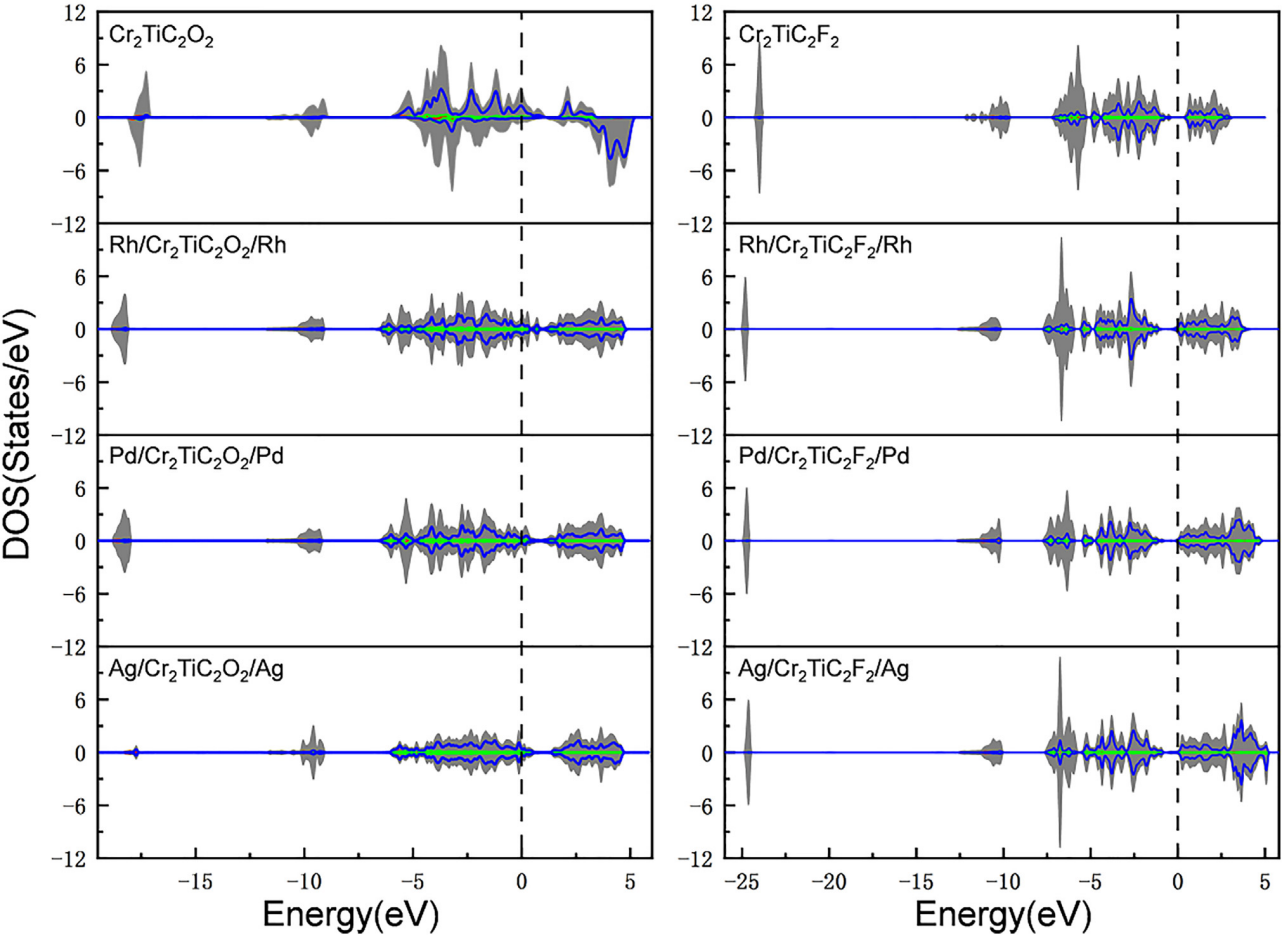
DOS of Cr_2_TiC_2_O_2_ and Cr_2_TiC_2_F_2_ in the M/Cr_2_TiC_2_O_2_/M and M/Cr_2_TiC_2_F_2_/M heterojunctions

The total density of states (DOS) of Cr_2_TiC_2_O_2_ and Cr_2_TiC_2_F_2_ in the M/Cr_2_TiC_2_T_x_/M heterojunctions was calculated for determining their electronic properties and promoting their development for spintronic applications. In general, as the interaction between noble metals and Cr_2_TiC_2_T_x_ MXene is moderate (for Cr_2_TiC_2_O_2_) or relatively weak (for Cr_2_TiC_2_F_2_), the variation in the electronic structure is limited, as shown in Figure 7. For the M/Cr_2_TiC_2_O_2_/M heterojunctions, the DOS around the Fermi level is in the range of 2–6 states/eV, which means that these systems are excellent conductors. The M/Cr_2_TiC_2_F_2_/M heterojunctions retain clear semiconductor characteristics, especially for M = Pd or Ag. Thus, the electronic transport characteristics vary with the Cr_2_TiC_2_O_n/4_F_2−n/4_ terminations in the heterojunctions.

Compared with the strong influence that the adhesion of the Rh layers has on the magnetic properties of Cr_2_TiC_2_T_x_, the effect of these layers on the electronic transport properties is considerably less pronounced. Regardless of the inhomogeneous O coverage, it can be a single-layer A-type AFM material under the adhesion of Rh layers, while the conductive ability can be tuned by the oxygen coverage. The structure of Cr_2_TiC_2_T_x_ MXene is similar to that of bilayer CrI_3_, both of which have two Cr atomic layers. Differently, the Néel temperature of Rh/Cr_2_TiC_2_T_x_/Rh MXene ranges between 5∼110K depending on the F contents, while for bilayer CrI_3_ it is 31 K.^34^ In Cr_2_TiC_2_T_x_ MXene, there are strong metallic bonds between the two Cr atomic layers, making the structure relatively stable, whereas in bilayer CrI_3_, the layers are held together by van der Waals forces. Besides, the extra electrons can induce FM-AFM transition for Cr_2_TiC_2_T_x_ MXene. If it will become experimentally possible to manipulate the terminations of Cr_2_TiC_2_T_x_, it is envisaged that this single-layer A-type AFM material will have promising application in spin electronics.

### Conclusions

In summary, first-principles calculations were conducted to investigate the magnetic and electronic properties of M/Cr_2_TiC_2_T_x_/M heterojunctions. It was found that pure Cr_2_TiC_2_T_x_ prefers an FM arrangement for a high O coverage (>75%), while it prefers an AFM arrangement for a low O coverage (<60%). Thus, the non-uniform distribution of the O terminations on the Cr_2_TiC_2_T_x_ surfaces causes this material to be in a spin disordered state. However, the Rh layers adhered to the Cr_2_TiC_2_T_x_ surfaces can change this spin disorder to long-range spin order by donating electrons to the O terminations, transforming Cr_2_TiC_2_T_x_ into a single-layer A-type antiferromagnet. As the proportion of F termination increases from 0 to 100%, the exchange coupling constant J_1_ of the compound escalates from 0.5 to 15.9 meV. Concurrently, the Néel temperature experiences a significant rise from 8 K to 110 K. The DOS analysis shows that Cr_2_TiC_2_T_x_ exhibits excellent conductivity with O termination and semiconductor characteristics with F termination. These unique features make Cr_2_TiC_2_T_x_ a promising magnetic material for application in spin electronics.

### Limitations of the study

This investigation utilized first-principles calculations to demonstrate that the adhesion of Rh layers onto Cr_2_TiC_2_T_x_ surfaces can induce a transition from a spin-disordered state to long-range spin order through electron donation to the O terminations, thereby converting Cr_2_TiC_2_T_x_ into a single-layer A-type antiferromagnet. Nonetheless, this study is not without its limitations that warrant further attention. First, the reliance on first-principles methods means that the complexity of the model may not be fully captured, underscoring the necessity for additional experimental corroboration. Second, the assessment of the Néel temperature is relatively low, just 110 K. The challenge of enhancing this Néel temperature, or alternatively, the strategic exploitation of this low-temperature attribute in the design of spintronic devices, presents a pivotal direction for future research endeavors.

## STAR★Methods

### Key resources table

**Table:** 

REAGENT or RESOURCE	SOURCE	IDENTIFIER
**Software**		

VASP5.2	VASP team	https://www.vasp.at/

### Resource availability

#### Lead contact

Further information and requests for resources and reagents should be directed to and will be fulfilled by the lead contact, Jianhui Yang (huijiany@163.com).

#### Materials availability

This study did not generate new unique materials.

#### Data and code availability

Data reported in this paper will be shared by the lead contact upon request.
This paper does not report original codes.
Any additional information required to reanalyze the data reported in this paper is available from the lead contact upon request.

### Experimental model and study participant details

Our study does not use experimental models typical in the life sciences.

### Method details

Spin-polarized density functional theory was used for all calculations with the Vienna ab initio simulation package (VASP).^35^^,^^36^ Projector augmented wave (PAW) potentials were used for all calculations.^37^ The Perdew–Burke–Ernzerhof (PBE) functional was used to determine the exchange-correlation energy.^38^ The GGA + U approach was also used to model the Cr and Ti 3days electrons, and the effective U value for the Ti and Cr atoms was 3.1 and 4.1 eV, respectively.^39^ The kinetic-energy cutoff was set to 500 eV. The convergence criterion for self-consistent calculations is 10^−5^ eV. Ionic relaxation was performed until the force acting on each atom was below 0.01 eV/Å 5 × 5×1 k-point meshes were chosen using the Monkhorst–Pack method.^40^ The calculated Cr–Cr and Cr–C bond lengths were 3.08 and 2.18 Å for pure Cr_2_TiC_2_O_1.25_F_0.75_, which represents a mismatch of about 3.3% with the experimental results obtained for Cr_2_TiC_2_O_1.3_F_0.8_.^29^ The calculated band gap of Cr_2_TiC_2_F_2_ was 1.1 eV, which is close to the Heyd–Scuseria–Ernzerhof (HSE06) hybrid functional results.^20^ These findings indicate that our calculation methods are reasonable.

### Quantification and statistical analysis

Our study does not include statistical analysis or quantification.

## References

[1] Wadley P., Howells B., Železný J., Andrews C., Hills V., Campion R.P., Novák V., Olejník K., Maccherozzi F., Dhesi S.S. (2016). Electrical switching of an antiferromagnet. Science.

[2] Lebrun R., Ross A., Bender S.A., Qaiumzadeh A., Baldrati L., Cramer J., Brataas A., Duine R.A., Kläui M. (2018). Tunable long-distance spin transport in a crystalline antiferromagnetic iron oxide. Nature.

[3] Marti X., Fina I., Frontera C., Liu J., Wadley P., He Q., Paull R.J., Clarkson J.D., Kudrnovský J., Turek I. (2014). Room-temperature antiferromagnetic memory resistor. Nat. Mater..

[4] Jungwirth T., Marti X., Wadley P., Wunderlich J. (2016). Antiferromagnetic spintronics. Nat. Nanotechnol..

[5] He J., Ding G., Zhong C., Li S., Li D., Zhang G. (2019). Cr2TiC2-based double MXenes: novel 2D bipolar antiferromagnetic semiconductor with gate-controllable spin orientation toward antiferromagnetic spintronics. Nanoscale.

[6] Chen X., Zheng C., Zhang Y., Zhou S., Liu Y., Zhang Z. (2021). Identification and manipulation of spin wave polarizations in perpendicularly magnetized synthetic antiferromagnets. New J. Phys..

[7] Zheng C., Chen X., Zhou S., Liu Y. (2023). Terahertz magnetic excitation in antiferromagnets: atomistic spin simulations versus a coupled pendulum model. J. Phys. Condens. Matter.

[8] Münzenberg M. (2021). High-speed spins. Nat. Phys..

[9] Hortensius J.R., Afanasiev D., Matthiesen M., Leenders R., Citro R., Kimel A.V., Mikhaylovskiy R.V., Ivanov B.A., Caviglia A.D. (2021). Coherent spin-wave transport in an antiferromagnet. Nat. Phys..

[10] Vaidya P., Morley S.A., van Tol J., Liu Y., Cheng R., Brataas A., Lederman D., del Barco E. (2020). Subterahertz spin pumping from an insulating antiferromagnet. Science.

[11] Bai H., Zhang Y.C., Han L., Zhou Y.J., Pan F., Song C. (2022). Antiferromagnetism: An efficient and controllable spin source. Appl. Phys. Rev..

[12] Gao A., Liu Y.F., Hu C., Qiu J.X., Tzschaschel C., Ghosh B., Ho S.C., Bérubé D., Chen R., Sun H. (2021). Layer Hall effect in a 2D topological axion antiferromagnet. Nature.

[13] Tsai H., Higo T., Kondou K., Nomoto T., Sakai A., Kobayashi A., Nakano T., Yakushiji K., Arita R., Miwa S. (2020). Electrical manipulation of a topological antiferromagnetic state. Nature.

[14] Nair N.L., Maniv E., John C., Doyle S., Orenstein J., Analytis J.G. (2020). Electrical switching in a magnetically intercalated transition metal dichalcogenide. Nat. Mater..

[15] Yan H., Feng Z., Qin P., Zhou X., Guo H., Wang X., Chen H., Zhang X., Wu H., Jiang C., Liu Z. (2020). Electric-Field-Controlled Antiferromagnetic Spintronic Devices. Adv. Mater..

[16] Gong C., Zhang X. (2019). Two-dimensional magnetic crystals and emergent heterostructure devices. Science.

[17] Yang J., Zhang S., Li L., Wang A., Zhong Z., Chen L. (2019). Rationally designed high-performance spin-filter based on two-dimensional half-metal Cr2NO2. Matter.

[18] Jiang P., Kang L., Hao H., Zheng X., Zeng Z., Sanvito S. (2020). Ferroelectric control of electron half-metallicity in A-type antiferromagnets and its application to nonvolatile memory devices. Phys. Rev. B.

[19] Jiang P., Tao X., Hao H., Liu Y., Zheng X., Zeng Z. (2021). Two-dimensional centrosymmetrical antiferromagnets for spin photogalvanic devices. NPJ Quantum Inf..

[20] Yang J., Zhou X., Luo X., Zhang S., Chen L. (2016). Tunable electronic and magnetic properties of Cr2M'C2T2 (M' = Ti or V; T=O, OH or F). Appl. Phys. Lett..

[21] Jing Z., Wang H., Feng X., Xiao B., Ding Y., Wu K., Cheng Y. (2019). Superior Thermoelectric Performance of Ordered Double Transition Metal MXenes: Cr2TiC2T2 (T = -OH or -F). J. Phys. Chem. Lett..

[22] Yang J., Zhang S., Wang A., Wang R., Wang C.-K., Zhang G.-P., Chen L. (2018). High magnetoresistance in ultra-thin two-dimensional Cr-based MXenes. Nanoscale.

[23] Gomonay O., Baltz V., Brataas A., Tserkovnyak Y. (2018). Antiferromagnetic spin textures and dynamics. Nat. Phys..

[24] García-Romeral N., Morales-García Á., Viñes F., Moreira I.d.P.R., Illas F. (2023). Theoretical Analysis of Magnetic Coupling in the TiC Bare MXene. J. Phys. Chem. C.

[25] García-Romeral N., Morales-García Á., Viñes F., Moreira I.d.P.R., Illas F. (2023). How does thickness affect magnetic coupling in Ti-based MXenes. Phys. Chem. Chem. Phys..

[26] Yang X., Ding N., Chen J., Wang Z., An M., Dong S. (2023). Electrical tuning of robust layered antiferromagnetism in MXene monolayer. Appl. Phys. Lett..

[27] Hantanasirisakul K., Anasori B., Nemsak S., Hart J.L., Wu J., Yang Y., Chopdekar R.V., Shafer P., May A.F., Moon E.J. (2020). Evidence of a magnetic transition in atomically thin Cr2TiC2Tx MXene. Nanoscale Horiz..

[28] Zhang S., Zhou Y., Liang X., Wang Y., Wang T., Yang J., Lv L. (2022). Tuning the Magnetic Properties of Cr2TiC2Tx through Surface Terminations: A Theoretical Study. Nanomaterials.

[29] Hart J.L., Hantanasirisakul K., Lang A.C., Li Y., Mehmood F., Pachter R., Frenkel A.I., Gogotsi Y., Taheri M.L. (2021). Multimodal Spectroscopic Study of Surface Termination Evolution in Cr2TiC2Tx MXene. Adv. Mater. Interfac..

[30] Yang J., Luo X., Zhou X., Zhang S., Liu J., Xie Y., Lv L., Chen L. (2017). Tuning magnetic properties of Cr2M2C3T2 (M = Ti and V) using extensile strain. Comput. Mater. Sci..

[31] Liu L., Chen S., Lin Z., Zhang X. (2020). A Symmetry-Breaking Phase in Two-Dimensional FeTe2 with Ferromagnetism above Room Temperature. J. Phys. Chem. Lett..

[32] Jiang S., Li L., Wang Z., Mak K.F., Shan J. (2018). Controlling magnetism in 2D CrI3 by electrostatic doping. Nat. Nanotechnol..

[33] Anderson P.W. (1950). Antiferromagnetism. Theory of superexchange interaction. Phys. Rev..

[34] Lei C., Chittari B.L., Nomura K., Banerjee N., Jung J., MacDonald A.H. (2021). Magnetoelectric response of antiferromagnetic CrI3 bilayers. Nano Lett..

[35] Kresse G., Furthmüller J. (1996). Efficient iterative schemes for ab initio total-energy calculations using a plane-wave basis set. Phys. Rev. B.

[36] Kresse G., Hafner J. (1994). Ab-initio molecular-dynamics simulation of the liquid-metal amorphous semiconductor transition in germanium. Phys. Rev. B.

[37] Blöchl P. (1994). Projector augmented-wave method. Phys. Rev. B.

[38] Perdew J.P., Burke K., Ernzerhof M. (1997). Generalized gradient approximation made simple. Phys. Rev. Lett..

[39] Anisimov V.I., Aryasetiawan F., Lichtenstein A.I. (1997). First-principles calculations of the electronic structure and spectra of strongly correlated systems: the LDA C U method. J. Phys. Condens. Matter.

[40] Monkhorst H.J., Pack J.D. (1976). Special points for brillouin-zone integrations. Phys. Rev. B.

